# ‘Monster… -omics’: on segmentation, re-segmentation, and vertebrae formation in amphibians and other vertebrates

**DOI:** 10.1186/1742-9994-10-17

**Published:** 2013-04-11

**Authors:** David Buckley, Viktor Molnár, Gábor Németh, Örs Petneházy, Judit Vörös

**Affiliations:** 1 Museo Nacional de Ciencias Naturales MNCN–CSIC, c/José Gutiérrez Abascal 2, Madrid, 28006, Spain; 2 Budapest Zoo and Botanical Garden, Állatkerti krt. 6-12, Budapest, 1146, Hungary; 3 Mediso Medical Imaging, Alsótörökvész u. 14, Budapest, 1022, Hungary; 4 Kaposvár University, Guba Sándor u. 40, Kaposvár, 7400, Hungary; 5 Dpt. of Zoology Hungarian Natural History Museum, Baross u. 13, Budapest, 1088, Hungary

**Keywords:** Development, Evo-devo, Morphology, Osteology, Vertebrae

## Abstract

**Background:**

The axial skeleton is one of the defining evolutionary landmarks of vertebrates. How this structure develops and how it has evolved in the different vertebrate lineages is, however, a matter of debate. Vertebrae and vertebral structures are derived from the embryonic somites, although the mechanisms of development are different between lineages.

**Discussion:**

Using the anecdotal description of a teratological newt (*Triturus dobrogicus*) with an unusual malformation in its axial skeleton, we review, compare, and discuss the development of vertebral structures and, in particular, the development of centra from somitic cellular domains in different vertebrate groups. Vertebrae development through re-segmentation of the somitic sclerotomal cells is considered the general mechanism among vertebrates, which has been generalized from studies in amniotic model organisms. The prevalence of this mechanism among anamniotes is, however, controversial. We propose alternative developmental mechanisms for vertebrae formation that should be experimentally tested.

**Summary:**

Research in model organisms, especially amniotes, is laying the foundations for a thorough understanding of the mechanisms of development of the axial skeleton in vertebrates, foundations that should expand the extent of future comparative studies. Although immersed in the ‘-omics’ era, we emphasize the need for an integrative and organismal approach in evolutionary developmental biology for a better understanding of the causal role of development in the evolution of morphological diversity in nature.

## Background

Teratologies recurrently occur in all animal groups. Although generally considered incidental natural curiosities, they reflect intrinsic properties of developmental systems. Not every imaginable ‘monster’ is possible: similar to non-aberrant morphological variants, teratologies appear in nature in a discrete manner. If all of the normal and aberrant shapes found in a group were plotted in a geometrical space, thus constructing a theoretical ‘morphospace’, the resulting points would not be scattered all along the plot but would concentrate around specific areas. These areas would be defined by the characteristics of the developmental system of the group. This idea of monsters as ‘logical developmental entities’ is rooted in classic morphological [1] and modern developmental [2] studies. Teratologies are not random: they result from the truncation or alteration of specific developmental pathways. Therefore, the study of these alterations may reveal veiled or cryptic underlying processes involved in the generation of form [3]. In this study, we use the anecdotal description of the axial skeleton of a teratological individual of the Danube crested newt (*Triturus dobrogicus*) to review and discuss the developmental processes of segmentation, re-segmentation, and vertebrae formation in vertebrate lineages. Vertebrae development through re-segmentation of the somitic cellular domains is considered the general mechanism among vertebrates, but its occurrence in anamniotes is controversial. We propose alternative mechanisms of vertebrae development that should be experimentally tested in anamniotes. Furthermore, in this ‘-omics’ era, we stress the need for an organismal approach in evolutionary and developmental biology to better understand the causal role of development in the evolution of morphological diversity in nature.

### Segmentation, somites, and vertebrae formation

**Figure 1:**
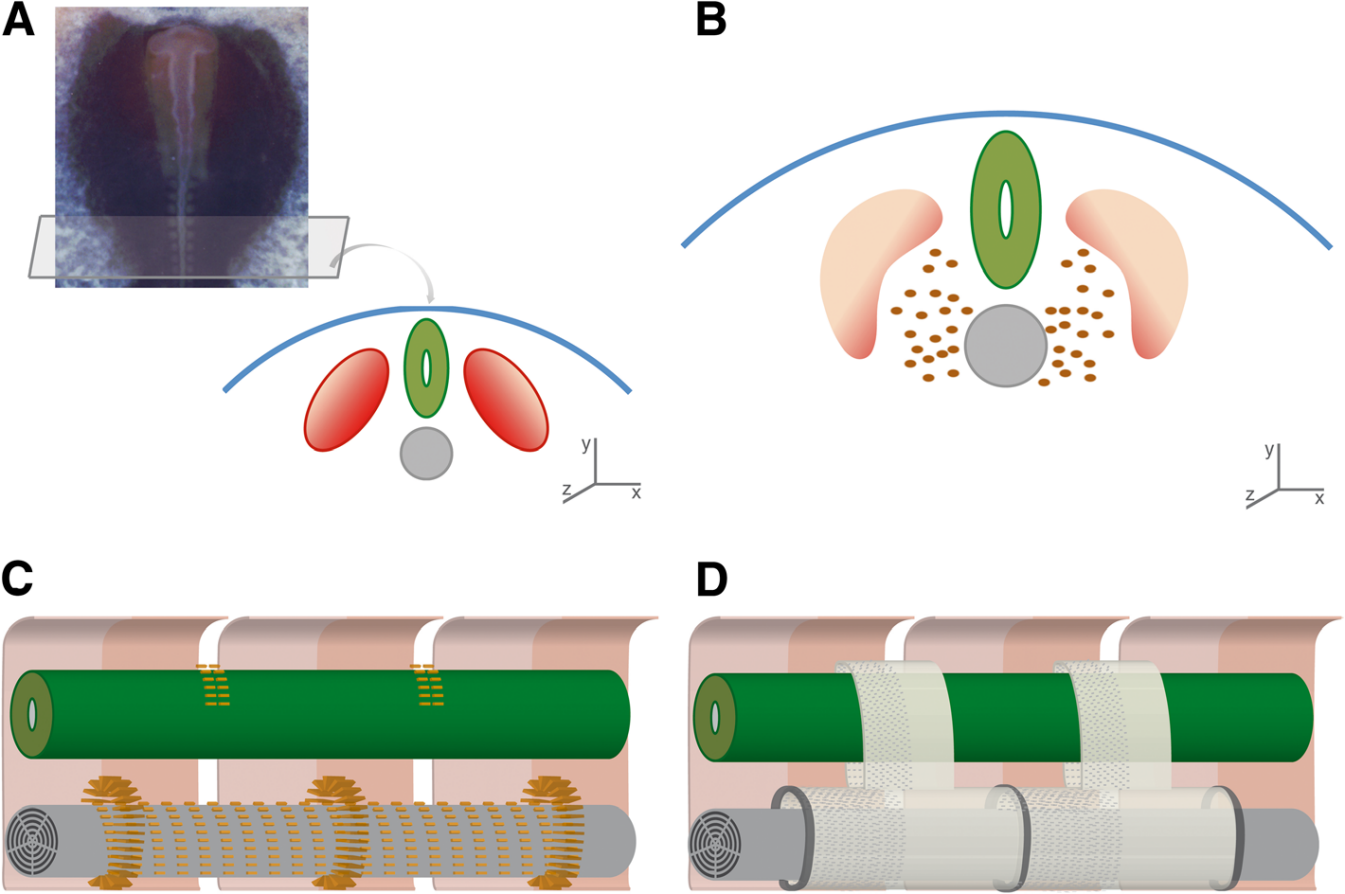
**Schematic representation of segmentation, re-segmentation, and vertebrae development in amniotes.** (**A**) Embryo of a chick (*Gallus gallus domesticus*), around 40 h post-fertilization. Note the rounded somites developing bilaterally to the neural tube. We also represent a scheme of a transversal section, showing the paired somites (red), surrounding the neural tube (green) and notochord (grey), under the dorsal ectoderm (blue). X, Y, Z: lateral, dorsal, and anterior axes, respectively. (**B**) Somites are regionalized and polarized soon after their formation. The dorsolateral area (light pink) constitutes the dermomyotome, differentiating eventually into axial muscles and dermis. The ventromedial area forms the sclerotome (orange), cellular precursor of vertebral skeletal elements. Sclerotomal cells delaminate from somites and migrate ventrally towards the notochord, and dorsally towards the neural tube. (**C**) Scheme of a longitudinal section (anterior to the left), showing three somites (pink) and their boundaries. Polarization of somites results in a rostral-caudal differentiation of the cellular domains (light and dark pink). Schematized is the initial migration of sclerotomal cells (orange), forming an unsegmented layer around the notochord, and perichordal rings at the level of the middle of each somite. Sclerotomal cells also aggregate dorsally, developing eventually into the neural arches. (**D**) Diagram of two chondrified vertebrae, with centra enveloping the notochord, and neural arches surrounding the neural tube. Intervertebral discs develop from the perichordal rings in (**C**), and centra and neural arches from the cells that migrate from the caudal part of the sclerotome of one somite (shaded) and the rostral part of the sclerotome of the adjacent somite (not shaded). Thus, vertebrae form at the intersomitic boundaries from cells from two adjacent somites. Vertebrae do not reflect the embryonic primary segmentation (somite position), but are displaced half a segment, a phenomenon known as re-segmentation. Chick picture is a courtesy of Sophie Miller.

Segmentation in vertebrates is revealed through the serial development of vertebrae and their related structures, such as ribs, vertebral apophyses, and axial muscles, all of which develop from the embryonic paired somites (Figure 1A). Somites form from the pre-somitic mesoderm (PMS) during embryogenesis. These paired structures form rostrocaudally along the embryonic neural axis, through the action of a ‘segmentation-clock oscillator’ that is regulated by several signaling pathways [4,5]. The pace and rhythm of the ‘segmentation-clock’ determine the final number of somites and, hence, the final number of vertebrae, which is a lineage- or species-specific trait [6]. After maturation and differentiation, somites generate different structures; the developmental fate of each somite (e.g., cervical, lumbar, or sacral vertebrae) is primarily specified by the early differential expression of *Hox* genes even prior to somite formation [7-9]. The highly regulated temporal and spatial expression of these genes leads to greater regionalization of the axial skeleton, thus shaping the possible morphological outcomes. Although highly conserved in evolution, *Hox* gene clusters have a characteristic number and arrangement that is lineage-dependent [10]. Segmentation and regionalization, thus, are two of the most important developmental processes involved in the formation of the axial skeleton in vertebrates and are responsible for the phenotypic variations and adaptions observed among lineages e.g., [11-14].

Developing embryonic somites, however, are not homogeneous structures. Following differentiation, they become polarized and compartmentalized, leading to several cellular domains that develop into different axial elements (e.g. muscles, ribs, rib bearers, centra) [15]. The dorsolateral half of the somites, for instance, forms the dermomyotome, which eventually differentiates into dermis and muscles. A space or cavity, called the myocoel, separates this domain from the ventromedial half of the somites. Cells from this later area form the sclerotome, which is the cellular precursor of the vertebral elements such as the centra, ribs and rib bearers. The sclerotome is also rostrocaudally polarized, with the two cellular sclerotomic subdomains being separated by the so-called sclerocoel.

As a general mechanism among vertebrates, sclerotome cells delaminate from the somites and form a thin unsegmented layer around the notochord (the perichordal tube) (Figure 1B) [16]. These cells then aggregate and form perichordal rings around the notochord approximately at the level of the middle of each somite (Figure 1C). Sclerotome cells also aggregate at somites boundaries either (i) dorsally, along the sides of the neural tube, eventually developing into vertebral neural arches, or (ii) ventromedially, forming the rudiments of the distal part of the ribs. Perichordal rings give rise to the intervertebral joints, and the area between them forms the centra (vertebral bodies), which align with the intersegmental neural arch rudiments. Therefore, neural arches, centra, and the distal part of the ribs develop at the intersomitic boundaries, but from different sclerotomal cell precursors and at different times.

The formation of centra differs among vertebrate lineages. In amniotes, for instance, centra, which form at somite boundaries, result from the migration of cells from the caudal half of one sclerotome and the rostral half of the sclerotome of the adjacent somite (Figure 1C,D) [4]. Therefore, amniotic vertebrae do not reflect the primary segmentation pattern (i.e., original somite location); rather, they are located intersegmentally and result from the redistribution of sclerotomal cells in a process known as re-segmentation.

The genetic control of segmentation and re-segmentation has been extensively studied in amniotes such as mouse and chick, leading to the general view that similar processes occur in all vertebrate lineages, which is not necessarily true. Although vertebrae are located intersegmentally in all vertebrates, it is unclear whether the active migration of cells from adjacent somites to form the centra is general among lineages. Re-segmentation in anamniotes is controversial. In some teleosts, a ‘leaky’ re-segmentation occurs in which centra are formed from cells originated from several somites [17]. Furthermore, the development of centra in other teleost lineages is strongly influenced by molecular signaling and cellular contribution from the notochord [18,19]. In fact, five different modes of centra formation have been described for teleosts [20].

In amphibians, somitogenesis has been thoroughly characterized in classic embryological studies [16,21], but the cellular and morphogenetic processes and the genetic and molecular mechanisms involved during amphibian vertebral segmentation are largely unknown for this group (with the exception of *Xenopus* and *Ambystoma*). Moreover, the evidence for re-segmentation in amphibians is equivocal. The sclerotome in salamanders and frogs is apparently reduced in cell number and not polarized rostro-caudally. Furthermore, no clear presence of sclerocoel has been shown in these two groups. The scant sclerotomal cell population forms late during development, and the developmental fate of these cells is unclear. It has been suggested, however, that there is no active movement of cells from adjacent sclerotomes to form the vertebrae centra [22-24]. In limbless amphibians (Gymnophiona), nevertheless, re-segmentation has been reported. In this case, the sclerotome is more developed and rostro-caudally polarized in two halves, separated by a sclerocoel; re-segmentation may have independently evolved in this lineage to distribute this larger cell population [25].

From the anecdotic observation of a rather unusual vertebral malformation in an individual of the newt *Triturus dobrogicus* (Amphibia, Caudata, Salamandridae), we discuss what is known (or assumed) about the developmental mechanisms and evolutionary patterns underlying vertebrae formation in the different vertebrate lineages.

## Discussion

### Insights from the ‘monster’

**Figure 2:**
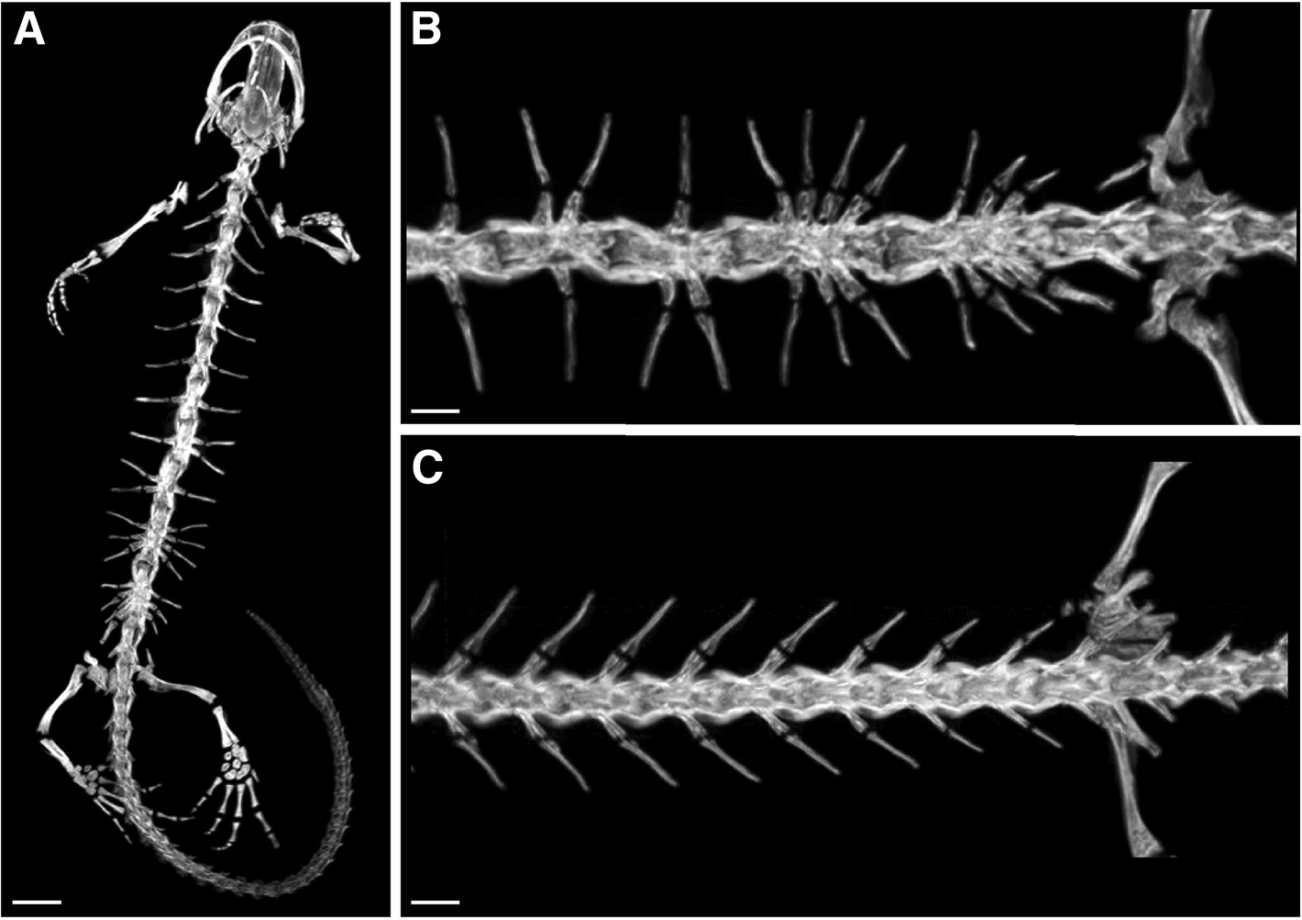
**Scans of the teratological newt *Triturus dobrogicus.*** (**A**) General overview of the skeleton. The teratological individual presents 17-paired ribs distributed in 11 vertebral bodies, plus an extra rib in the sacral region. (**B**) Detail of a normal *T*. *dobrogicus* specimen in dorsal view, showing the regular vertebral shape and location of ribs. (**C**) Dorsal view of the teratological specimen, showing the amalgamation of centra and the asymmetric distribution of ribs. The teratological specimen was collected in Velence, Pest County, Hungary (15/03/1967) by P. Bohn and placed in the Herpetology Collection of the Hungarian Natural History Museum (catalogue nr. 67.12.1.). The normal individual was collected in Kiskunhalas, Bács-Kiskun County, Hungary (21-23/03/1975) and placed in the Herpetology Collection of the Hungarian Natural History Museum (catalogue nr. 57.41.1.). The scans were taken with a NanoSPECT/CTTM In vivo pre-clinical imager (Bioscan Inc., Washington DC, US, manufactured by Mediso, Budapest, Hungary) on 55 kVp tube voltage, 0.145 mA tube current, and 1500 ms exposure time. The images were reconstructed with an exact cone beam method and with 50x50x50 μm3 voxel size. We used the software InVivoScope™ with maximum intensity projection to visualize the image. Scale bar in A = 0.5 mm; scale bars in B and C = 0.1 mm.

**Figure 3:**
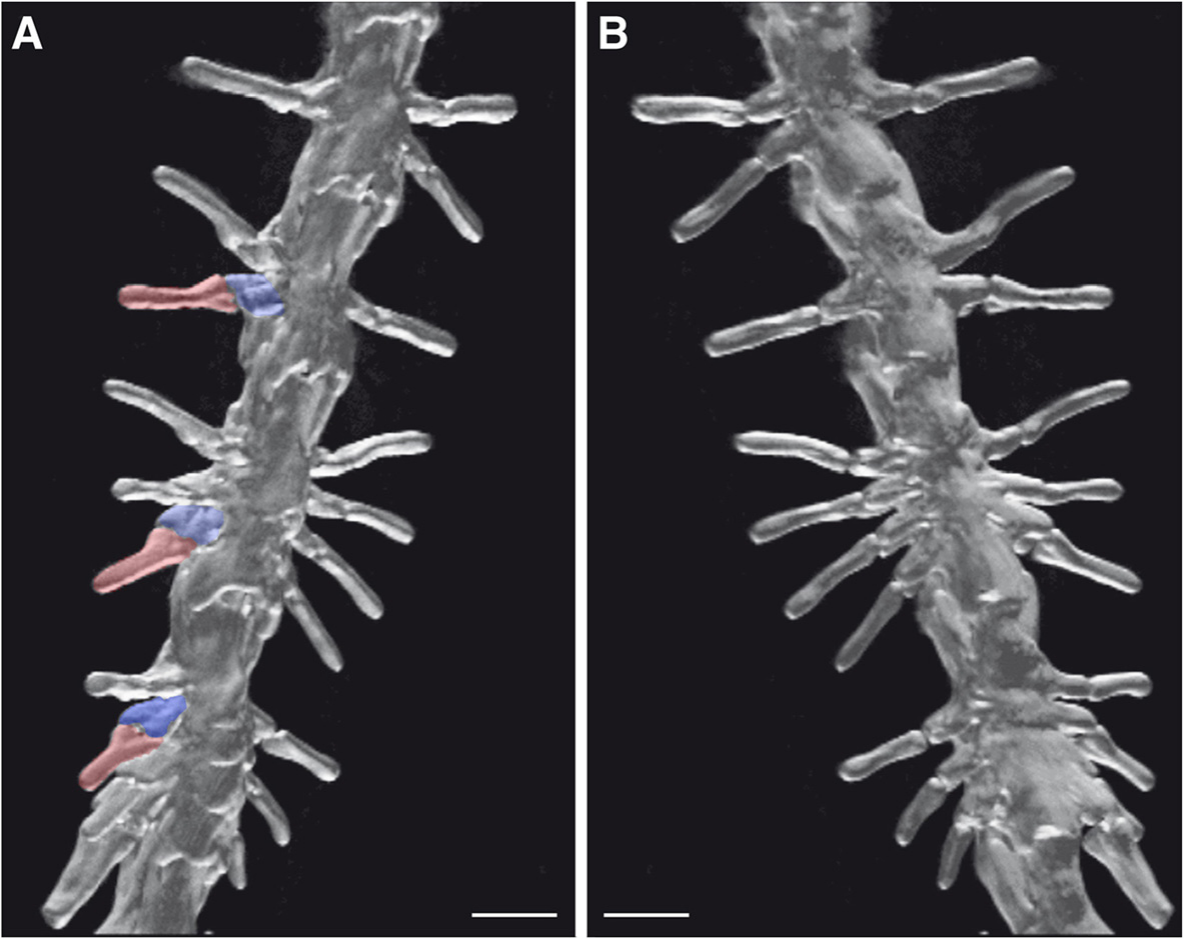
**Details of the teratological *Triturus dobrogicus *specimen in a dorsal (A) and ventral (B) view.** Note that the bicapitate ribs (in red) and rib-bearers (in blue) are clearly visible in spite of the fusion of vertebrae. Neural arches are also visible, although amalgamated. Scale bars = 0.2 mm.

**Figure 4:**
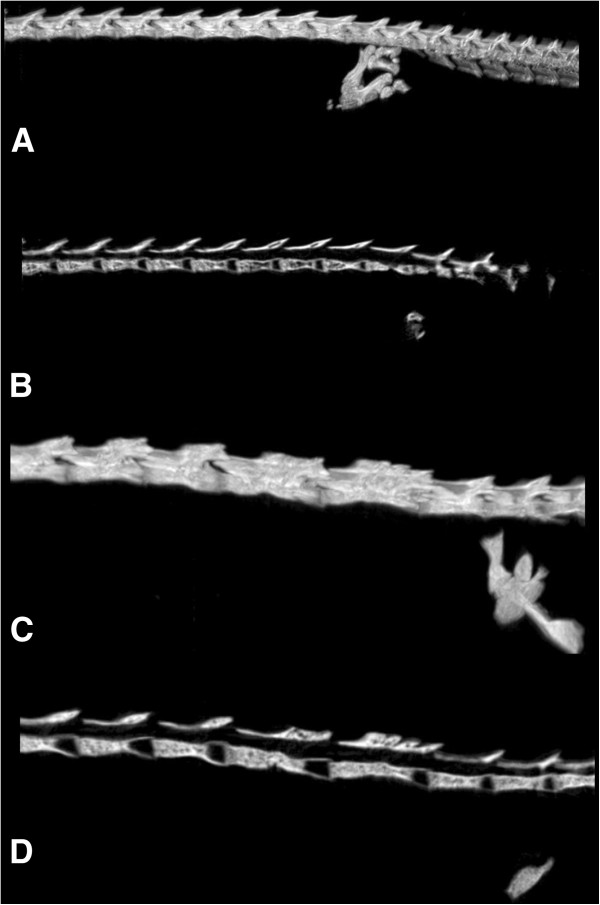
**Details of the normal (A, B) and teratological (C, D) specimens of *T. dobrogicus *in lateral view (anterior to the left), showing the distribution of centra and neural arches in the pelvic region. B** and **D** are the sagittal sections of the same views. In **D**, we can observe four amalgamated neural arches developed on a single enlarged or fused vertebral centrum.

During a survey of morphological variability in populations of the newt *Triturus dobrogicus* in the Herpetological Collection of the Hungarian Natural History Museum, a teratological adult was found (Figure 2A,C). Normal *T*. *dobrogicus* individuals have 17 or 18 rib-bearing vertebrae, including the sacral vertebra (Figure 2B) [26]. The teratological specimen has 17-paired ribs, distributed asymmetrically in 11 vertebral bodies, plus an extra rib in the sacral region (Figure 2A,C). What should correspond to the last nine trunk vertebrae plus the sacral appear in the teratological specimen as four ‘fused’ or enlarged vertebral bodies, bearing the corresponding ribs (10 pairs) but distributed asymmetrically across centra. All of the vertebral elements (ribs, rib-bearers, and neural arches), however, can be recognized in the fused or enlarged vertebral centra (Figures 3 and 4). Given that vertebral elements arise from different sclerotomal cellular precursors and develop under different genetic controls (although coordinated through common signaling pathways) [27], this suggests that somites and the sclerotomal cell precursors of the vertebral elements (i.e., primary segmentation) were present and properly specified in the teratological newt. However, the process of secondary segmentation (i.e., intersegmental location of centra) would have been disrupted in this individual.

The causal origin of the malformation is difficult to assess. This is a rather unusual phenotype, especially concerning the asymmetric distribution of ribs in the fused or enlarged centra, which, as far as we know, has not been previously reported in the literature. To our knowledge, no mutant studies have revealed similar phenotypes or malformations like the one described here. The teratology could have a genetic or epigenetic nature and thus related either to mutations or to the exposure of exogenous teratogenic agents during development, respectively [28]. Several genetic pathways have been shown to be critical during somite and vertebrae development. *Hox* genes are known to be directly involved in somite and vertebrae formation and, thus, could be involved in the teratology. *Hox* gene mutations and mis-expression, however, lead most frequently to homeotic transformations, which are not observed this case. Mutations in regulatory and signaling genes have also been related to various vertebral syndromes and aberrant phenotypes [29,30]. For example, the signaling molecule retinoic acid (RA) is involved in left-right symmetry of somite formation [31]. The disruption of the RA signaling pathway may lead to various syndromes involving symmetry defects on the vertebral axis, which could also be related to the pattern observed in this study.

However, given the correct specification of ribs and other axial elements, vertebrae anomalies of the teratological specimen are more likely to be a consequence of an abnormal process during later developmental stages rather than due to defects during early segmentation (e.g., *Hox* gene mis-expression). Furthermore, and more importantly, we argue that, regardless of the primary cause of the teratology, the resulting phenotype is a consequence of the specific properties of vertebrae formation in urodeles. Amalgamations of centra, coupled with the complete development of apophyses and the asymmetric distribution of ribs and rib bearers, reflect specific developmental processes during vertebrae formation in urodeles. Specifically, the asymmetric pattern observed here could be easily explained if an active process of cell movement, i.e. re-segmentation, occurred in urodeles, which has been suggested to be not the case although this has not experimentally proved or refuted [22]. It could also be the case, however, that the somite boundaries and the movement of the re-segmented sclerotomal cell population do not specify centrum limits in urodeles: other molecular or mechanical mechanisms could specify the correct location of vertebral bodies (secondary segmentation) such as, for example, the specification of intervertebral joints by the notochord (see below). In this case, the four abnormally enlarged vertebral bodies could be the result of the incorrect molecular/cellular/physical specification of the centra limits, yet coupled to the correct primary segmentation specification (somites and vertebral structures). Are there unknown developmental mechanisms involved in vertebrae formation in amphibians? As little recent experimental work has been done exploring vertebral development in amphibians, these questions remain unresolved. The study of this teratological newt does not provide definitive answers to these questions either, although it suggests some possibilities and some non-mutually exclusive experimental lines that should be further explored in amphibians and other non-model vertebrate lineages:

(i) Interestingly, the anomaly reported here occurs in the last trunk vertebrae. It has been suggested that the anterior trunk region is much more conservative in terms of potential variability than the posterior part of the axis [22]. Furthermore, in a population survey of the plethodontid urodele *Batrachoseps*, Jockusch [32] reported a high incidence of developmental abnormalities in vertebrae at the thoracic/sacral region, including asymmetric pelvis articulations and insertion of one or more extra half vertebrae on only one side of the vertebral axis. Furthermore, the final number of trunk vertebrae was not specified until late in development. This has also been shown for other salamander species that have the capacity to add segments to their tails (i.e., vertebrae) post-embryonically, at adult stages [33]. The number of embryonic somites, thus, would not specify the final number of vertebrae. This is interesting since it has been show that in some teleosts that the trunk and tail somites form differently during gastrulation. The transition zone between these two regions corresponds, furthermore, to the area of the axial skeleton presenting the highest levels of morphological variability [17]. This might indicate that there are different genetic, cellular, and developmental mechanisms involved in the differential development of the vertebral structures along the vertebral axis, a hypothesis that should be further explored in all vertebrate lineages.

(ii) Although not morphologically segmented, the notochord may present a cryptic segmental pattern through spatial and temporal differential gene expression. It is known, for instance, that notochord expression of *Shh* and *Noggin* drives the differentiation of the ventral somite into the sclerotome and sclerotome proliferation [34,35]. Moreover, in fused somite mutants of zebrafish, it has been shown that the sclerotomal polarization is disrupted. The segmentation of the sclerotome-derived neural arches is abnormal although the vertebral centra are correctly specified, which is opposite to the pattern observed in this study. The authors interpret their results as a primordial role of the nothochord in specifying segmental identity and position [36]. The notochord may also represent an ancestrally segmented structure in vertebrates that specified the regular disposition of perichordal rings and, hence, the intersegmental location of centra. Indeed, in some teleosts, the segmental expression of alkaline phosphatase activity (ALP) in the notochord is related to the initial ossification of centra suggesting an active role for the notochord during segmentation [37]. This and related possibilities should be tested in all vertebrate lineages.

(iii) The spinal cord may also play an important role in vertebrae differentiation. It has been shown through ablation studies, for instance, that the spinal cord influences the development of the neural arches in the urodeles *Ambystoma* and *Taricha*[38-40]; however, the molecular mechanisms involved are unknown at present. Furthermore, the elements of the peripheral nervous system (PNS, neural crest cells and outgrowing motor and sensory neurons) have to migrate out the central nervous system in every segment of the axial skeleton. It has been shown that these elements follow specific movement patterns that are directly related to the anterior/posterior polarization of the somites: they migrate along their anterior parts since they are ‘repulsed’ by molecules at the posterior halves [41]. It has been suggested that in anamniotes, given the scarcity of the sclerotomal cellular population, it is the dermomyotome that drives the repulsion/migration pattern [41]. It remains to be tested then, what are the specific roles of the spinal cord and the paired ganglia present in each segment, if they play any significant function in specifying the intersegmental location of vertebral bodies, and the potential relation between the migrating elements of the PNS, the migrating sclerotomic cells, and the dermomyotome [42].

(iv) Molecular cell-labeling markers for sclerotomal cells that have recently been developed for the axolotl *Ambystoma mexicanum*[43] may permit a detailed analysis of the cellular origin of vertebral centra in this amphibian, such that the contribution of each somitic sclerotomal cellular population to each vertebral element could be discerned. Furthermore, cell-labeling experiments of notochord cells would also shed light on the role of the notochord as it is known that notochord cells may contribute to the formation of centra in certain lineages such as in some teleosts [18] and urodeles [22,38]; this apparently does not happen in other lineages (e.g., mammals) [44].

Overall, these proposed lines of research will help determine whether re-segmentation occurs in urodeles and anurans. In addition, they will help to identify the exact cellular origin of centra in amphibians and the tissues and structures involved in vertebral differentiation. More importantly, the study and comparison in an explicit phylogenetic framework of the morphological variations, and their developmental and molecular underpinnings, will provide a better understanding of the homologous and homoplastic elements are responsible for the evolution of the axial skeleton among the particular vertebrates lineages.

## Summary

### Expanding the analytical framework

The axial skeleton is often considered an anatomical module, a morphological unit that defines and characterizes the vertebrate lineage. However, the genetic, developmental, and historical origin of each vertebral element may be different. The different axial components have not evolved at the same geological time [35], but were only eventually integrated into the functional anatomical module that we observe today. A longstanding debate, for instance, has revolved around the homology of centra in (stem and crown) fossil vertebrates and the modern fishes and tetrapods (e.g., [21,45,46]). Most of the comparative studies have approached this question either form a developmental or paleontological perspective, although neither approach has satisfactorily addressed it. Furthermore, accepted views of evolution of axial elements in early tetrapods have been recently challenged by new paleontological findings, which require reinterpreting embryological and developmental evolutionary inferences. For instance, it has been shown that specialization and regionalization of the axial skeleton was already present in rhipidistian fishes, which were though to represent un-regionalized ancestral forms [47]. In the same vein, reverse patterns of ossification in the diplospondylous stem-tetrapod *Ichthyostega* have been recently reported [48], which could bring a new perspective concerning the levels of plasticity and developmental axial patterns in tetrapod ancestral forms. Understanding how the different axial components evolved into a genetically, developmentally, ecologically, and evolutionary cohesive structure, therefore, is a major challenge that has to be faced from a multidisciplinary perspective. Evolutionary developmental biology, or evo-devo, would be an appropriate integrative framework for such a study.

Evolutionary developmental biology seeks to explain morphological evolution in a causal context by studying how development generates evolutionary phenotypic variants and how developmental mechanisms evolve over time. Although broad in scope, the bulk of current research in evo-devo is quite specialized and reductionist in its approach, driven mainly by research programs in developmental genetics in model organisms. However, new technical advances have made it more feasible for evo-devo research both in non–model organisms and at the population level. This unusual teratological non-model individual exemplifies the necessity to frame evo-devo research programs at the interface of development, natural history, population variability, and phylogenetics, including the extinct taxa. Much is known about development and evolution of vertebrae but, still, the unusual malformation reported here cannot be explained from a developmental point of view, since the referred mechanisms and developmental processes have been generalized from studies in lineages with different developmental properties and evolutionary histories. Research in model organisms, especially amniotes, is laying the foundations for a thorough understanding of the mechanisms of development of the axial skeleton in vertebrates. These foundations, however, should not be use to infer common general ‘laws’ for the evolution of the axial skeleton in vertebrates, but to establish the basis for further comparative analyses. Major theoretical contributions of evo-devo would be derived from a comprehensive understanding of the causal links between developmental properties, which includes, but is not restricted to, the genetic and molecular toolkit, and the intraspecific variability of these properties in natural populations, followed by a interspecific (phylogenetic) comparison across a wide range of extinct and extant evolutionary lineages. Therefore, in this “-omics” era, we emphasize an integrative and organismal approach in evolutionary developmental biology as an exceptional tool for the study of morphological diversity and evolution.

## Competing interests

The authors declare that they have no competing interests.

## Authors’ contributions

DB and JV conceived the idea of the manuscript. VM, GN and OP organized and performed the CT scans and DB and JV wrote the manuscript. All the authors read and approved the final version of the manuscript.
